# Sensitivity enhancement of a Cu (II) metal organic framework-acetylene black-based electrochemical sensor for ultrasensitive detection of imatinib in clinical samples

**DOI:** 10.3389/fchem.2023.1191075

**Published:** 2023-05-22

**Authors:** Xuanming Xu, Shun Li, Xingwei Luan, Chao Xuan, Peng Zhao, Tingting Zhou, Qingwu Tian, Deng Pan

**Affiliations:** Department of Clinical Laboratory, The Affiliated Hospital of Qingdao University, Qingdao, Shandong, China

**Keywords:** imatinib, metal-organic frameworks, acetylene black, electrochemical sensor, glassy carbon electrode

## Abstract

Imatinib (IMB), an anticancer drug, is extensively used for chemotherapy to improve the quality of life of cancer patients. The aim of therapeutic drug monitoring (TDM) is to guide and evaluate the medicinal therapy, and then optimize the clinical effect of individual dosing regimens. In this work, a highly sensitive and selective electrochemical sensor based on glassy carbon electrode (GCE) modified with acetylene black (AB) and a Cu (II) metal organic framework (CuMOF) was developed to measure the concentration of IMB. CuMOF with preferable adsorbability and AB with excellent electrical conductivity functioned cooperatively to enhance the analytical determination of IMB. The modified electrodes were characterized using X-rays diffraction (XRD), X-ray photoelectron spectroscopy (XPS), fourier transform infrared (FT-IR), ultraviolet and visible spectrophotometry (UV-vis), electrochemical impedance spectroscopy (EIS), scanning electron microscopy (SEM), energy dispersive X‐ray spectroscopy (EDS), brunauer‒emmett‒teller (BET) and barrett‒joyner‒halenda (BJH) techniques. Analytical parameters such as the ratio of CuMOF to AB, dropping volumes, pH, scanning rate and accumulation time were investigated through cyclic voltammetry (CV). Under optimal conditions, the sensor exhibited an excellent electrocatalytic response for IMB detection, and two linear detection ranges were obatined of 2.5 nM-1.0 μM and 1.0–6.0 μM with a detection limit (DL) of 1.7 nM (S/N = 3). Finally, the good electroanalytical ability of CuMOF-AB/GCE sensor facilitated the successful determination of IMB in human serum samples. Due to its acceptable selectivity, repeatability and long-term stability, this sensor shows promising application prospects in the detection of IMB in clinical samples.

## 1 Introduction

Imatinib (IMB, also known by its trade name of Gleevec/Glivec), is a type of chemotherapy medication approved by the Food and Drug Administration (FDA) in 2001 (Diculescu et al., 2009). As a pioneer tyrosine kinase inhibitor (TKI), it is applied to treat certain types of cancers such as chronic myelogenous leukemia (CML) and acute lymphocytic leukemia (ALL), for patients in which the Philadelphia chromosome is positive (Ph^+^) (Qu et al., 2019). Moreover, imatinib also deregulates the tyrosine kinase activity of *c-Kit* associated with gastrointestinal stromal tumors (GISTs) (Gajski et al., 2019; Li et al., 2022). In addition, this orally administered drug is rapidly absorbed and its absolute bioavailability is generally almost 76% (Peng et al., 2005; Roosendaal et al., 2020). Thus, it can be mainly metabolized by the liver CYP3A4 enzyme into N-desmethyl imatinib, which has a bioactivity similar to that of its parent medicine (Adiwidjaja et al., 2020).

Therapeutic drug monitoring (TDM) refers to the clinical practice of measuring the drug concentrations in biological fluids of patients at specified time intervals and controlling their drug doses in a timely manner to formulate individualized dosing regimens (Mueller-Schoell et al., 2021). Current evidence from clinical trials shows that when the measured steady-state minimum plasma concentrations of IMB were (C_min,ss_) ≥1,000 ng/mL, complete cytogenic response (CcyR) and major molecular response (MMR) resulted in significant improvement in CML patients (Miura, 2015). However, a C_min,SS_ of IMB >3,000 ng/mL was associated with a higher incidence of adverse events (AEs) (Guilhot et al., 2012). Hence the effective therapeutic window of the C_min,SS_ of imatinib should be maintained between 1,000 and 3,000 ng/mL to achieve dose optimization in precise clinical treatment. Remarkably, concentrations of imatinib and its metabolite in plasma have demonstrated significant interindividual variation (Farag et al., 2017).

In the past few decades, various analytical techniques have been developed for IMB determination, such as fluorescence (Yan et al., 2016), liquid chromatography-tandem mass spectrometry (LC‒MS/MS) (Kralj et al., 2012), high-performance liquid chromatography (HPLC) (D’Avolio et al., 2012) and capillary electrophoresis (CE) (Rodríguez Flores et al., 2003). Among them, liquid chromatography and mass spectrometry have time-consuming pretreatment processes and require professionals with relevant knowledge backgrounds (Chen et al., 2020). Capillary electrophoresis frequently produces unreliable results with limited sensitivity (Sánchez-López et al., 2016), while the drawback of fluorescence is that it is generally vulnerable to interfering substances (Zheng et al., 2019). These shortcomings of the reported approaches have forced researchers to develop rapid and convenient methods. Determination methods based on electrochemical sensors have been extensively applied with a number of advantages, such as timeliness, no complex preprocessing procedures, real-time detection under *in situ* conditions, and highly sensitive and selective analysis of clinical samples (Feng et al., 2021; Wang et al., 2022). Moreover, the presence of electrical activity sites on the IMB surface means that an electrochemical detection method is feasible (Hassan Pour et al., 2021). Accordingly, it is of great significance to establish a simple, fast and low-cost IMB electrochemical detection method.

Metal organic frameworks (MOFs) are a type of porous coordination polymers (PCPs) that have multidimensional network structures, and are synthesized by combining inorganic metal ions or metal clusters with organic ligands (Xu et al., 2010; Zhou et al., 2015; Yola, 2021). By virtue of their expandable pore surfaces, multiple coordination sites and outstanding adsorption capacity, MOFs have recently received significant attention for application in catalysts, supercapacitors, and drug delivery, storage and separation (Zhou et al., 2015; Abbasi et al., 2016; Kumari et al., 2022). Despite these limitations, the application of MOFs in the field of electrochemistry still faces some obstacles, such as poor electrical conductivity and an unstable structure in aqueous environments (Kumari et al., 2022).

As one of the most extensively applied square-hole MOFs, CuMOF (Cu_3_(BTC)_2_ (BTC = 1,3,5-benzenetricarboxylate), also known as HKUST-1) is a potential adsorbent with a changeable large specific surface area, easily adjustable pore size and porous composition (Jahangiri–Dehaghani et al., 2022; Wachholz Junior et al., 2022). Additionally, CuMOFs can be suitable for applications that require the frequent loading and unloading of guests, and the activated empty phase of CuMOF has preferable affinity for IMB through hydrogen bonding (Abbasi et al., 2016). Due to these properties, CuMOF is a good candidate for IMB determination.

Acetylene black (AB), as a special class of porous carbon black, is obtained from acetylene (C_2_H_2_) which is decomposed exothermically under pressure during oxygen-free conditions (Yang et al., 2014). It is a perfect nonmetallic electrocatalyst due to its superior electrical conductivity, large specific surface area and enhanced electronic transfer efficiency (Xu et al., 2010; Feng et al., 2021).

**SCHEME 1:**
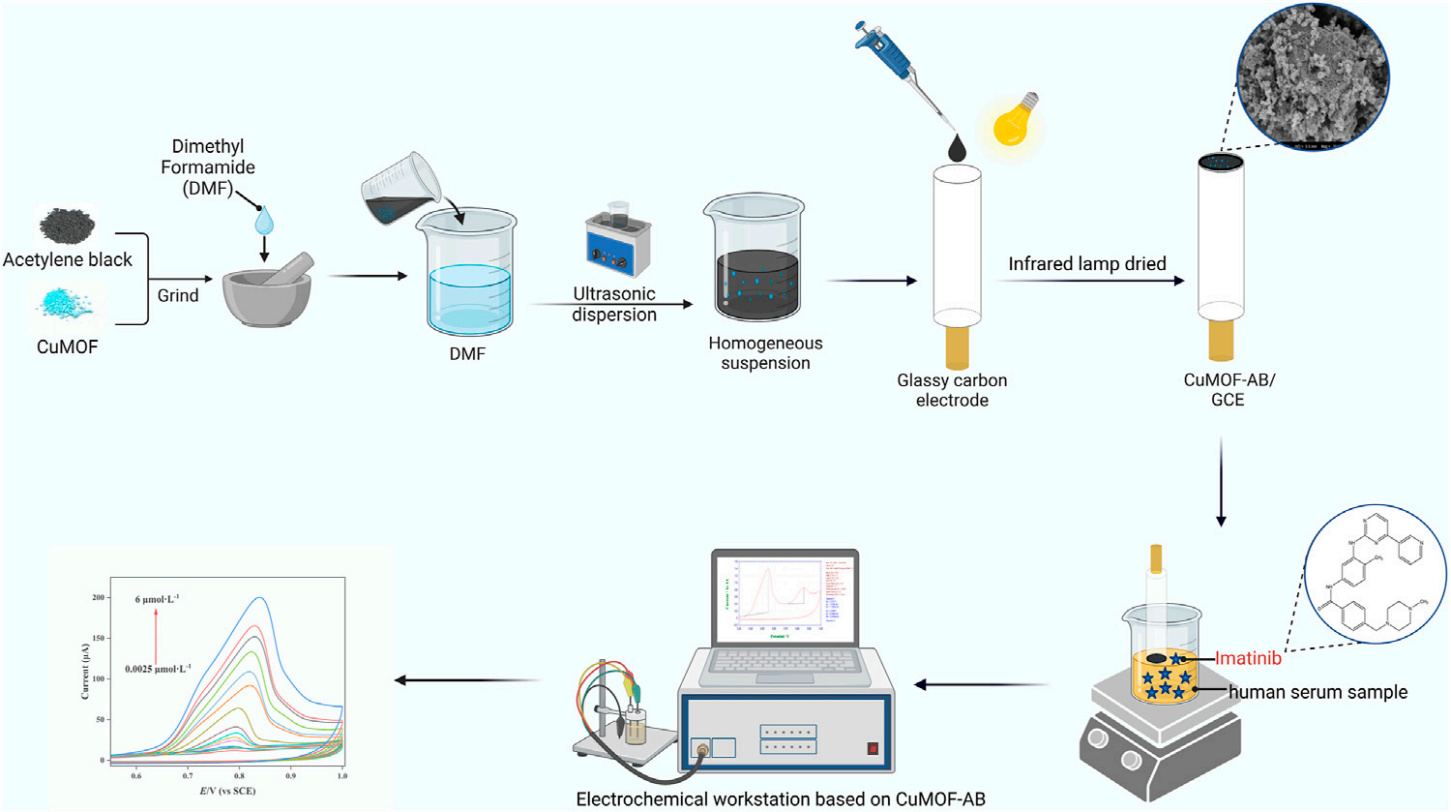
Modification steps of the proposed CuMOF-AB/GCE sensor. CuMOF-AB was cast on the surface of GCE. An electrochemical workstation consisting of a saturated calomel electrode, platinum (Pt) pillar electrode and modified GCE, was applied for the enhanced detection of IMB in human serum samples.

On the basis of the above analysis, materials with superior electrical conductivity are generally used to improve the electron transfer capability of electrode surfaces modified by MOFs. Until now, combining CuMOF with AB for analytical application has rarely been reported. In this regard, a novel, efficient and lower LOD sensor was fabricated based on properties of AB and CuMOF structures due to excellent sensitivity, high selectivity and outstanding adsorptivity, for the electrochemical analysis of IMB in the present research (as shown in Scheme 1). In addition, the nano-materials were characterized by XRD, XPS, FT-IR, UV-vis, SEM, EDS, BET, and EIS methods. The synthesized materials were used to modify the surface of GCEs and successfully applied for IMB concentration detection in real serum samples.

## 2 Experimental section

Materials and reagents, equipment, electrode preparation, optimization of procedure parameters and procedures of the real samples are described in the Supplementary Material.

## 3 Result and discussion

### 3.1 Characterization of the materials

**FIGURE 1:**
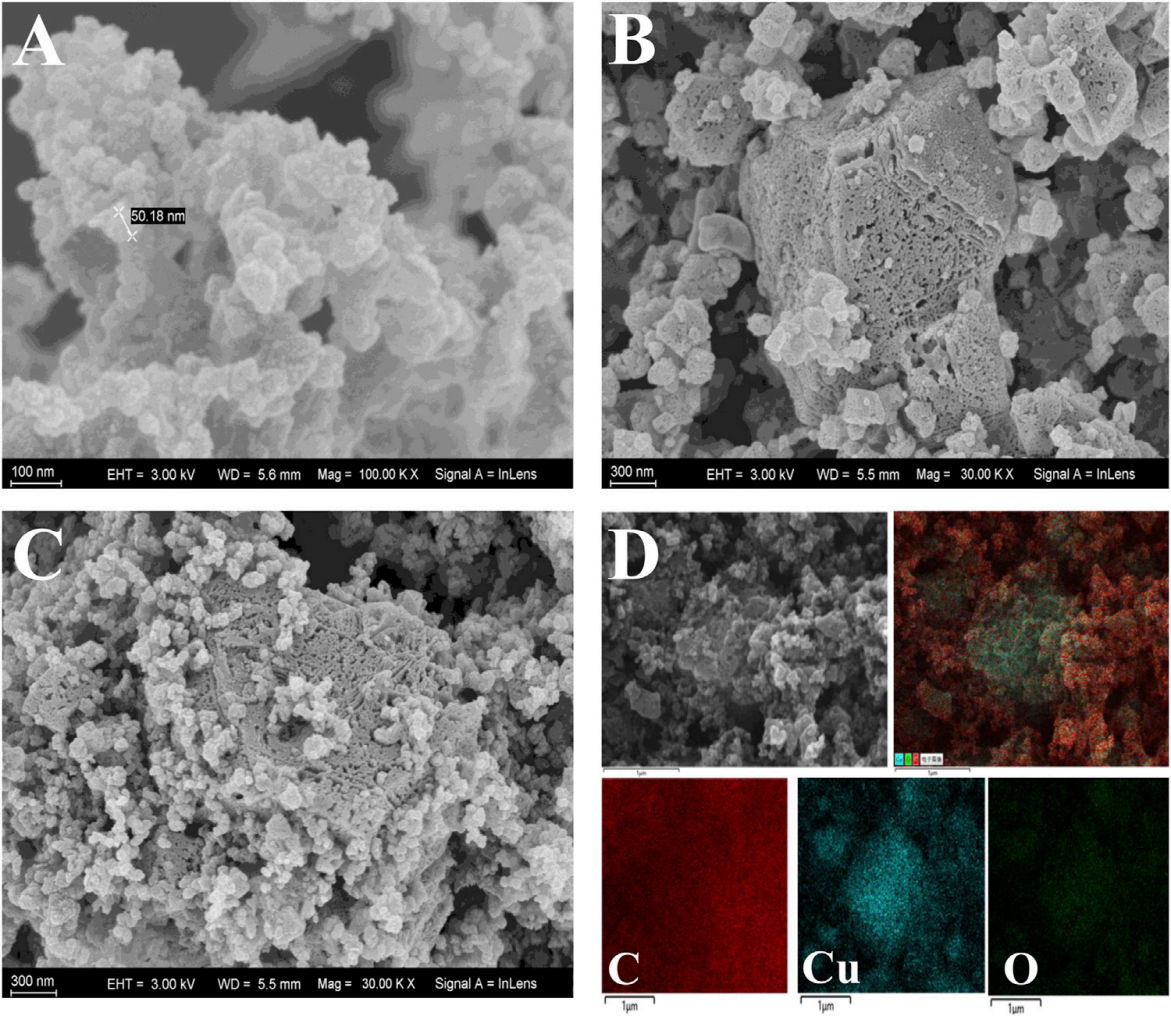
SEM images of AB **(A)**, CuMOF **(B)** and CuMOF-AB **(C)**. SEM mapping images of CuMOF-AB **(D)**.

The surface morphology and structure of AB, CuMOF and CuMOF-AB were evaluated using scanning electron microscopy (SEM) with a 20 kV accelerating voltage. As shown in Figure 1A, the particles of AB were spherical with an average diameter of 50 nm and had homogeneous surfaces with irregular polishing grooves; Figure 1B shows that the CuMOF crystals were octahedral in shape with distinct edges and sharp corners, and the various surface pore sizes enhanced the adsorption capacity. The SEM image shown in Figure 1C displays the morphology of the AB and CuMOF composites, and it was obvious that both materials were interpenetrated. In addition, in the SEM mapping image of CuMOF-AB (Figure 1D), carbon, oxygen and copper were found to be uniformly distributed throughout the nanocomposites. According to the energy dispersive spectroscopy (EDS) spectra of CuMOF (Supplementary Figure S1A), the C, O and Cu contents were 60.55%, 18.11% and 21.34%, respectively. However, from the results of the CuMOF-AB nanocomposite presented in Supplementary Figure S1B, the C content (83.10%) was obviously higher than the Cu and O contents, indicating the existence of AB.

**FIGURE 2:**
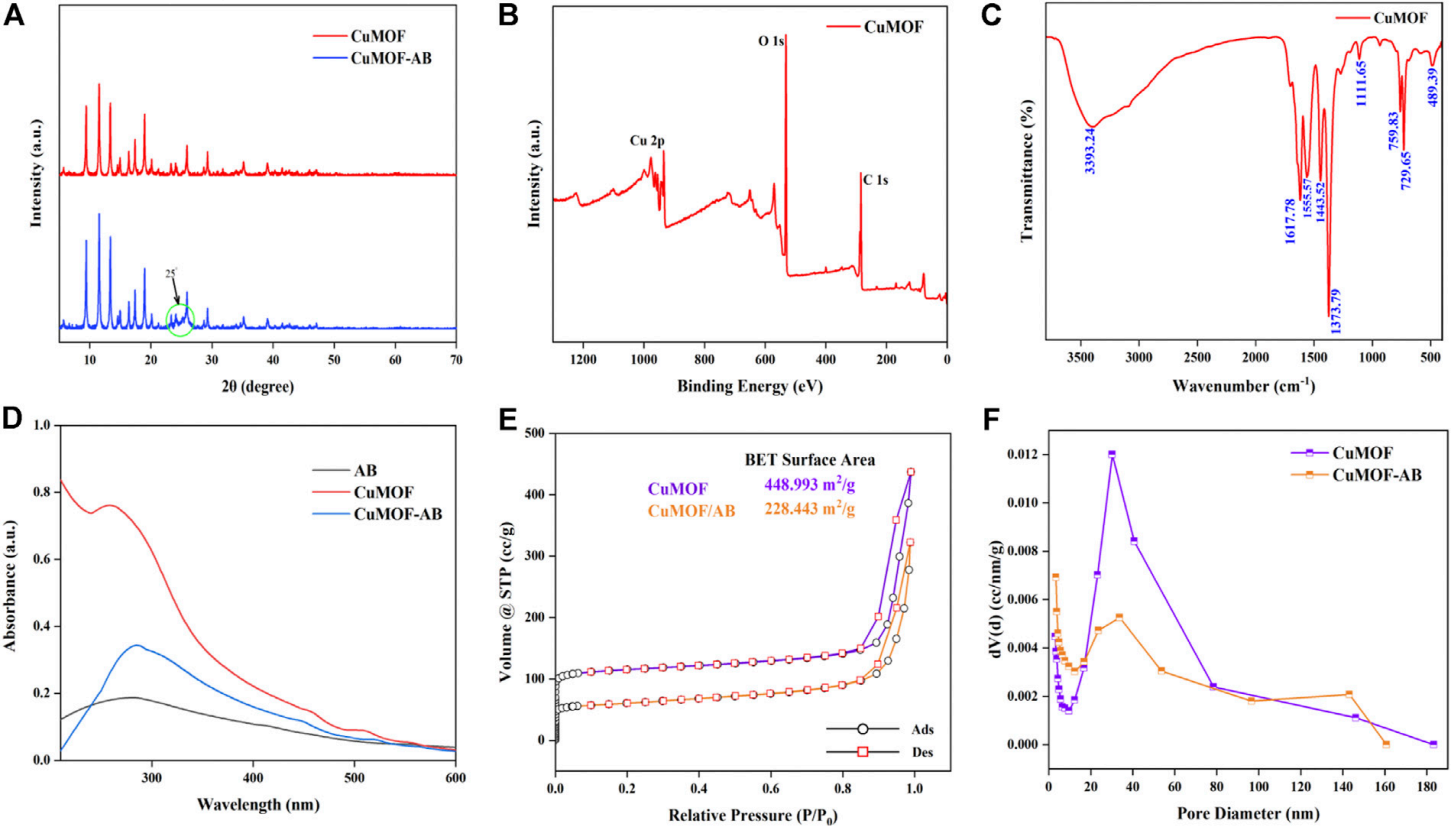
XRD **(A)** crystallography of the CuMOF and CuMOF-AB composites. XPS **(B)** and FT-IR **(C)** spectra of CuMOF. UV-visible spectroscopy **(D)** of AB, CuMOF and CuMOF-AB. Nitrogen adsorption−desorption isotherms **(E)** and pore diameter distribution **(F)** of CuMOF and CuMOF-AB.

The XRD patterns of CuMOF and CuMOF-AB composites had been provided in Figure 2A. CuMOF showed distinctive diffraction sharp peaks at 2θ of about 9.4^°^, 11.6^°^, 13.4^°^, 14.9^°^, 16.4^°^, 17.4^°^ and 19.0^°^ (Li et al., 2018; Rezvani Jalal et al., 2020), and the high peak intensities also could be readily confirmed the high crystalline structure of MOF. According to the previous literature, acetylene black only had a broad peak at 25^°^ due to its amorphous structure (Sun et al., 2015), so the width of the diffraction peaks at 25^°^ increased obviously in the composites.

XPS analysis was used to determine the chemical and electronic states. It could be demonstrated from the spectrum (Figure 2B) that the Cu, C and O elements coexist in CuMOF-AB. The characteristic peaks of C 1s and O 1s located at the binding energies of about 285 and 532 eV, respectively. In Cu 2p regions, Cu 2p_3/2_ peak at 935.1 eV and its shakeup satellites were associated with Cu (II). The peak appearing at 955.1 eV with a satellite at 963.1 eV were assigned to Cu 2p_1/2_ (Quan et al., 2021; Zhang et al., 2023).

Confirmation of the synthesis of CuMOF were fairly supported by the outcomes of FT-IR. Figure 2C showed the broad peak around 3393.24 cm^−1^ region were apparent which was assigned to OH bands vibration of intercalated water. The absorption peaks at 1,617, 1,555, and 1,373 cm^−1^ could be assigned to the characteristic vibrations of C=O, and the band at about 1,443 cm^−1^ was ascribed to the C=C stretching in the aromatic structure (Wang et al., 2013). In addition, the absorption bands at 950–600 cm^−1^ were owing to C-H bending of benzene ring (Abbasi et al., 2017).

CuMOF related characteristic absorbance peak was at about 258 nm in the ultraviolet-visible (UV-vis) patterns (Gao et al., 2023). And introduction of AB made the new absorption peak exhibited a positive shift from 258 to 284 nm, indicating that CuMOF-AB nanocomposites had successfully formed (Figure 2D).

The adsorption-desorption isotherms of CuMOF and CuMOF-AB are shown in Figure 2E, which conformed to a *Type I* isotherm according to the IUPAC classification, indicating the existence of mesopores. The dramatic uptake at extremely low P/P_0_ was due to the enhanced adsorptive interactions under narrow micropore conditions causing micropore filling (Thommes et al., 2015). Figure 2E shows that the BET specific surface areas of CuMOF and CuMOF-AB were 448.993 and 228.443 m^2^/g, and the pore volumes were 0.68 and 0.50 cc/g, respectively. The BJH technique was used to investigate the average pore diameters of CuMOF and CuMOF-AB, which were found to be 30.257 nm and 3.410 nm, respectively, by the adsorption method (Figure 2F). These results are summarized in Supplementary Table S1. Due to the incorporation of AB, it was clear that compared with CuMOF, the pore size of CuMOF-AB composites changed significantly, indicating that CuMOF-AB nanocomposites were successfully prepared.

### 3.2 Electrochemical characterization of the modified electrodes

The electroactive surface areas of the bare GCE, AB/GCE and CuMOF-AB/GCE modified electrodes were measured at various scan rates in solution containing 0.25 mM [Fe(CN)6]^3-/4-^ and 0.1 M KCl. Randles-Sevcik equation (Foroughi et al., 2021) was computed to analyze the efficacy of embedded sensor with bare GCE, AB/GCE and CuMOF-AB/GCE at 25°C.

$$I_{P}=269000\;n^{3/2}{AD}^{1/2}{Cv}^{1/2}$$ (1)

Where, *Ip* is the peak current, *n* shows the number of electrons consisted in the process (n = 1), *D* stands for the diffusion coefficient (7.6 × 10^−6^ cm^2^/s), *A* for the electrode surface area (cm^2^), *v* for the scan rate (V/s) and *C* for the [Fe(CN)6]^3-/4-^ concentration (mol/cm^3^) (Taherizadeh et al., 2023). As shown in Supplementary Figure S2, the slopes from the plot of current against scanning rate square root were used for the calculation of the electroactive surface areas, and *A* values were 0.08, 0.11, and 0.14 cm^2^ for the surfaces of bare GCE, AB/GCE and CuMOF-AB/GCE, respectively. So CuMOF-AB could be reinforced during electroanalysis because of an impressive elevation in its electroactive surface area.

EIS, as a powerful characterization technique, is used to measure the impedance of analyzed samples in the appropriate frequency range by applying a sinusoidal voltage or current. Moreover, the actually measured Nyquist spectra are always fitted with equivalent electrical circuits, which are regarded as electrical fingerprints, to evaluate electrochemical properties and behaviors. The high-frequency part of the Nyquist plot indicates charge transfer resistance (*R*_ct_), which is represented by the diameter of the semicircle. In contrast, in the low-frequency region, the straight lines correspond to ion diffusion in redox reactions. In addition, the change in *R*_ct_ value is determined by the characteristics of the modifiers and modification reaction on the surface (Singh et al., 2023).

**FIGURE 3:**
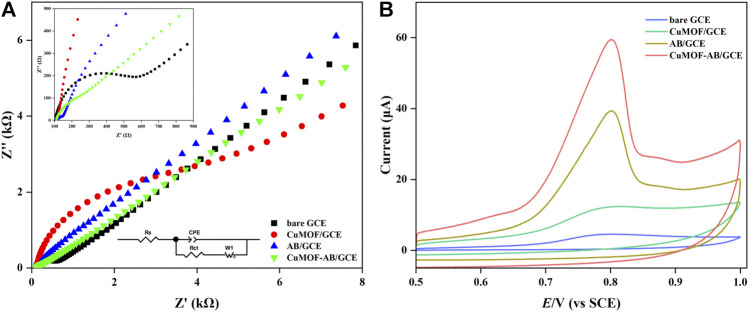
**(A)** Nyquist curves of bare GCE, CuMOF/GCE, AB/GCE and CuMOF-AB/GCE in 0.1 M KCl containing 5 mM K_3_Fe [(CN)_6_] and K_4_Fe [(CN)_6_] solution. The spectra were acquired at 50 mV ac amplitude in the frequency range from 100 kHz to 0.01 Hz **(B)** CVs of the bare GCE, CuMOF/GCE, AB/GCE and CuMOF-AB/GCE in 0.5 μΜ IMB solution (pH = 7) at a scanning rate of 100 mV/s.

The EIS equivalent circuit model of the four different electrodes is shown in Figure 3A (insets), where *R*_s_ and *W*_1_ are the electrical resistance of the electrolyte and the Warburg impedance, respectively. To improve accuracy, a constant phase element (CPE) is used to model *C*_dl_, which represents the capacitance of a double layer at the electrode and electrolyte (Grossi and Riccò, 2017). In addition, the Nyquist plot (Figure 3A) of the bare GCE showed a relatively large semicircle with an *R*_ct_ of 600 Ω. After the modification of the GCE with AB, the value of *R*_ct_ decreased to 66 Ω, which confirmed that the electron-transfer property of AB/GCE improved. For CuMOF/GCE, both the diameter and *R*_ct_ (6,456 Ω) increased, which demonstrated that the presence of CuMOF hindered electron transfer on the electrode surface, which might have occurred because the CuMOF framework is intrinsically insensitive to electrocatalysis. Compared with the bare GCE and CuMOF/GCE, CuMOF incorporated with AB exhibited additional decreases in *R*_ct_ (370 Ω). Consequently, it was inferred that AB compensated for the poor conductivity of the CuMOF and notably facilitated fast electron transfer between the [Fe(CN)_6_]^3−/4−^ solution and the modified GCE surface.

### 3.3 Electrochemical behavior of IMB on GCE

To investigate the electrochemical behavior of IMB (3 μΜ) on different modified electrodes, CV was used to record oxidation currents and potentials in 0.1 M PBS (pH = 7). As shown in Figure 3B, oxidation peaks were observed at approximately +0.82 V in the positive potential scanning, but the corresponding peaks of reduction were not observed in the reverse scanning. This phenomenon proved that the IMB reaction on all the above modified electrodes was not reversible. The current response of the bare GCE was barely noticeable, revealing that the electron transfer process was extremely slow on the GCE only. In contrast, for the AB-modified electrode and CuMOF-modified electrode, the signals of IMB oxidation were slightly higher than those of the bare electrode. The oxidation peak current of CuMOF-AB/GCE was the largest, indicating that the fabricated CuMOF-AB composite film had better electroanalytical performance. A possible reason for this was the excellent conductivity of AB and the high catalytic and adsorption ability of porous CuMOF, in which the interior pores had Lewis acid coordination copper sites that were readily accessible for catalytic conversions (Schlichte et al., 2004). The adsorption of IMB by CuMOF might be dominated by hydrogen bonding to secondary building units (SBUs) and subsequently to other IMB molecules (Abbasi et al., 2016). These observations elucidated that IMB on CuMOF-AB/GCE exhibited the best electrochemical behavior.

### 3.4 Optimization of the analytical parameters

To ensure optimal sensing performance, the mass ratio of CuMOF to AB, drop volume of CuMOF-AB on the GCE and accumulation time were studied. While holding other conditions equal, the oxidation peak current gradually increased when increasing the mass ratio from 0:1 to 1.5:1 and then started to decrease as the mass ratio continued to increase (Supplementary Figure S3). A possible explanation for this was that the binding force of hydrogen bonds between the MOFs and IMB was weak due to the lack of CuMOF at low ratios, but at high ratios, the modified electrodes had poor conductivity and impeded electron transfer on the GCE surface, which was attributed to the reduction in the relative content of AB. Therefore, 1.5:1 was selected as the best ratio in this test.

A certain dropping volume of CuMOF-AB was a major and meaningful factor in the test procedures and had to be optimized. The CuMOF-AB volume effect was evaluated ranging from 4 to 8 μL, and the peak current was monitored. As shown in Supplementary Figure S4, the signal of the modified electrode increased up to V_CuMOF-AB_ = 6 μL, and then the current values tended to be constant. These results indicated that the electrode surfaces were not fully covered by the prepared materials when the dropping volume was less than 6 μL, and then they reached a saturation state. Thus, considering material consumption and economic cost, 6 μL was selected as the optimal volume in further analysis. Additionally, the effect of the accumulation time (t_ac_) of IMB on the sensor response was also investigated. As illustrated in Supplementary Figure S5, the maximum value of the relevant current was procured within 7 min and reached an equilibrium state. IMB adsorption on the GCE achieved saturation after 7 min, so t_ac_ = 7 was chosen for subsequent analysis.

As a quadrivalent base, imatinib is pH-sensitive owing to the presence of an amine and a pyridine group (Qi et al., 2016). Similarly, because most organic compounds are susceptibly influenced by the solution pH value in oxidation reactions, the oxidation behaviors of 3 μM IMB were necessarily evaluated using the fabricated CuMOF-AB/GCE in 0.1 M PBS with the pH ranging from 3.0 to 9.0. As shown in Supplementary Figures S6 and S4A, the oxidation peak current increased from pH 3.0 to a maximum at pH 7.0 and then markedly decreased with further increases in pH; hence, this pH value was selected as the most appropriate pH. Due to the pK_a_ of IMB (pK_a1_ = 2.5, pK_a2_ = 4.0 and pK_a3_ = 8.2) (Mioduszewska et al., 2017), this drug was positively charged in solution at pH 7.0 and negatively charged at pH > 8.2. Moreover, because the surfaces of the CuMOF adsorbent were negatively charged when pH > 4 (Azhar et al., 2016), the maximum current was obtained (pH = 7) due to the electrostatic attraction of different charges. It should also be noted that the oxidation peak potentials shifted to more negative potentials as the pH increased, indicating H^+^ participation in the oxidation process of IMB. Furthermore, Rodríguez (Rodríguez et al., 2018) found that the loss of the two protons from piperazine rings occurred at pK_a_ values equal to approximately 4.0 and 8.0; hence, the IMB carried two positive charges when pH values were less than 4.0 and had only one positive charge until the pH was up to 8.0. As shown in Supplementary Figure S6, two distinct peaks were observed from pH 3 to 5, and one peak was observed at higher pH values. Moreover, the pH values were linearly correlated with *E*_P_ in the range of 3-8, and the linear equation was determined as *E*_P_ = −0.024 pH + 0.717 with *R* ^2^ = 0.993. The calculated slope was −0.024 (24 mV/pH), which was almost half of the theoretical Nernstian value (59 mV/pH) (Walczak et al., 1997), indicating that two electrons and one proton were involved in the IMB electro-oxidation process. These results were in accordance with those previously reported in the literature (Rodríguez et al., 2018; Rezvani Jalal et al., 2020; Hassan Pour et al., 2021). In addition, the line of *E*_P_ vs. pH was broken at approximately pH 8, which was almost coincident with the pK_a3_ value of IMB.

### 3.5 Electrochemical mechanism of IMB on the CuMOF-AB/GCE

**FIGURE 4:**
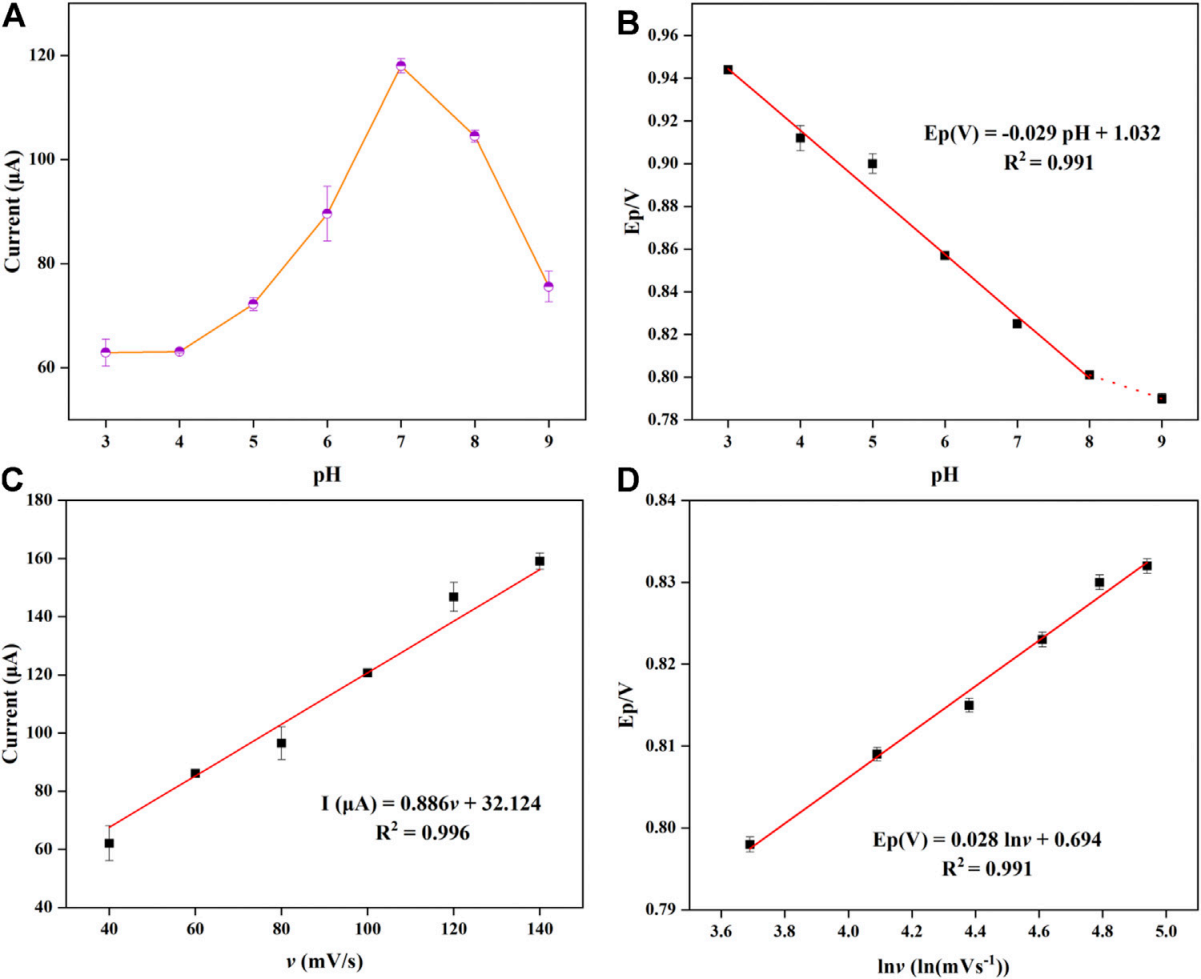
**(A)** Plots of the oxidation peak current vs. pH values from 3.0 to 9.0 at 100 mV/s. **(B)** Potential diagram of different pH values in the presence of 3 μM IMB in PBS solution. **(C)** Corresponding plot of current vs. scanning rate (40–140 mV/s) in the presence of 3 μΜ IMB in PBS solution. **(D)** Linear relationship between *E*_P_ and logarithm of scanning rate.

**SCHEME 2:**
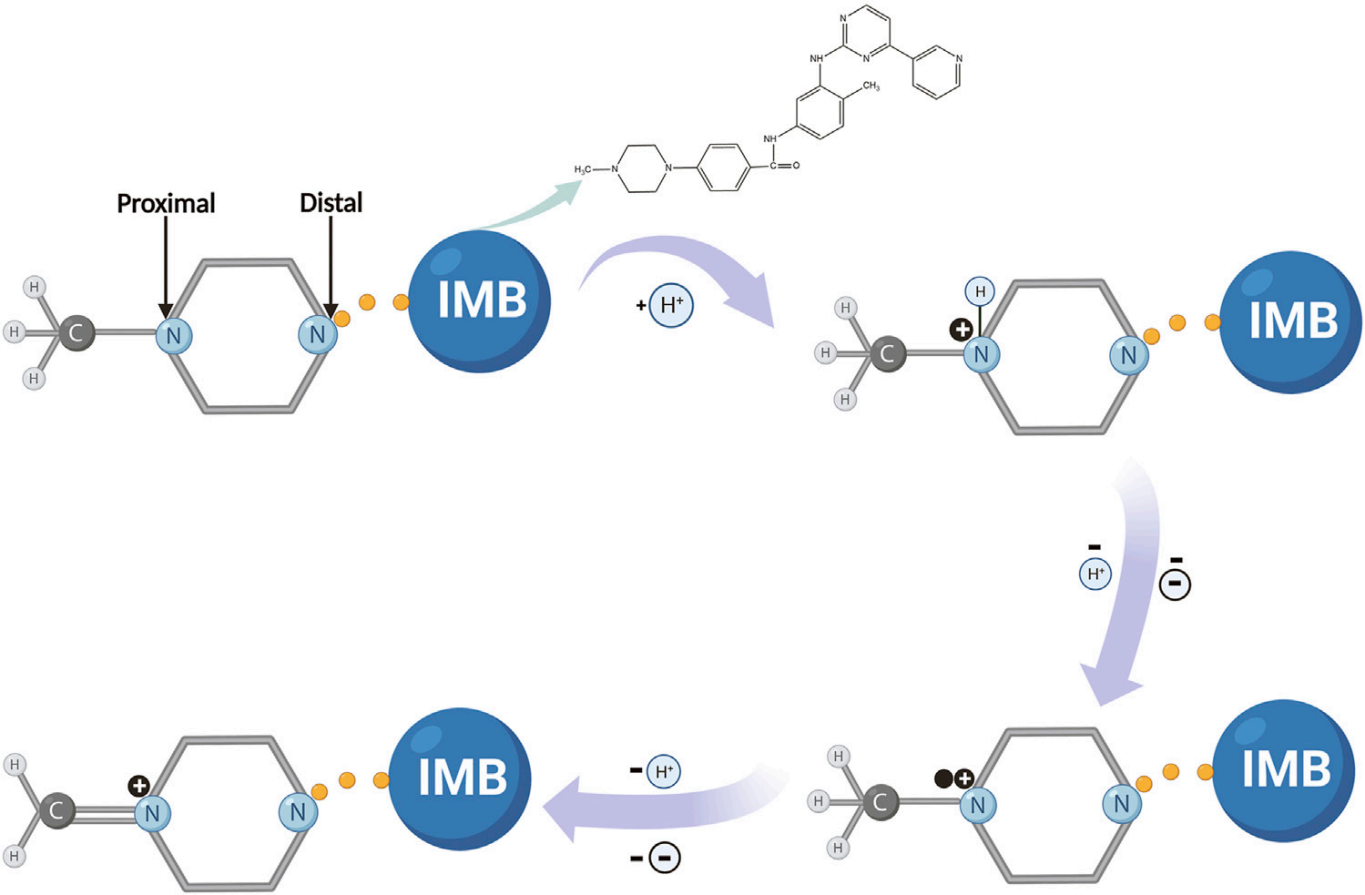
Possible reaction mechanism for IMB on the modified electrode.

To determine the mechanism of the electrochemical process of CuMOF-AB/GCE, the CV of CuMOF-AB/GCE was collected while varying the scanning rate from 40 to 140 mV/s, as shown in Supplementary Figure S7. Under the optimized conditions, the peak current (*I*_P_) and potential (*E*_P_) progressively moved to positive values as the scanning rate (*υ*) increased. As shown in Figure 4C, the peak current exhibited a great linear relationship with the scanning rates, and the corresponding regression equation was determined to be I (μA) = 0.886*υ* +32.124 (*R* ^2^ = 0.996), indicating an adsorption-controlled process of IMB transport on the electrode surface (Rodríguez et al., 2018). Additionally, the linearity (Figure 4D) between *E*_P_ and ln *υ* confirmed the intrinsic irreversibility of the IMB electrochemical processes and conformed to Laviron’s equation (Laviron, 1979), which is expressed as follows:

$$E_{P}{=E}^{0}{+[RT/\left(1-\alpha \right)\;nF]\;ln\;[RTk}_{s}/\left(1-\alpha \right)\;nF]+[RT/\left(1-\alpha \right)\;nF]\;ln\;\upsilon$$ (2)

where *E* ^0^ is the standard redox potential, *R* is the molecular gas constant (8.314 J mol^−1^ k^−1^), *T* is the Kelvin temperature [*T* (298.15 K) = 273.15 + 25°C], *F* is the Faraday constant (96485.333 C mol^−1^), *n* is the number of interchanged electrons, *k*_s_ is the rate constant of the electrochemical reaction, *α* is the charge-transfer coefficient, and *υ* is the various scanning rates. According to the slope (0.027) of *E*_P_ vs. ln *υ*, the value of (1*−α*) *n* was calculated to be equal to 0.94. Moreover, *α* was usually identified as 0.5 during the irreversible redox reaction; therefore, two electrons were involved in the oxidation of IMB on the surface of CuMOF-AB/GCE. This suggested conclusively that the electron transfer was consistent with that in the above PBS solution (pH = 7), so the possible electro-oxidation mechanism for IMB was proposed as shown in Scheme 2. In this overall mechanism, the nitrogen atom on the piperazine ring of IMB, as a pyridine compound molecule, undergoes electro-oxidation, accompanied by the removal of a proton and two electrons. The process is described as follows. First, the terminal nitrogen on the piperazine ring is protonated. Subsequently, another aliphatic nitrogen (i.e., proximal nitrogen) partially forms a cationic radical after losing a proton and an electron. In the next step, the piperazine moiety loses the same number of protons and electrons to convert into a quaternary Schiff base (Hammerich, 2003; Özkan et al., 2004; Uslu et al., 2005). Notably, this oxidation mechanism for IMB is almost consistent with that of the boron-doped diamond electrode reported by Brycht et al. (Brycht et al., 2016).

### 3.6 Analytical characteristics of the modified electrode

**FIGURE 5:**
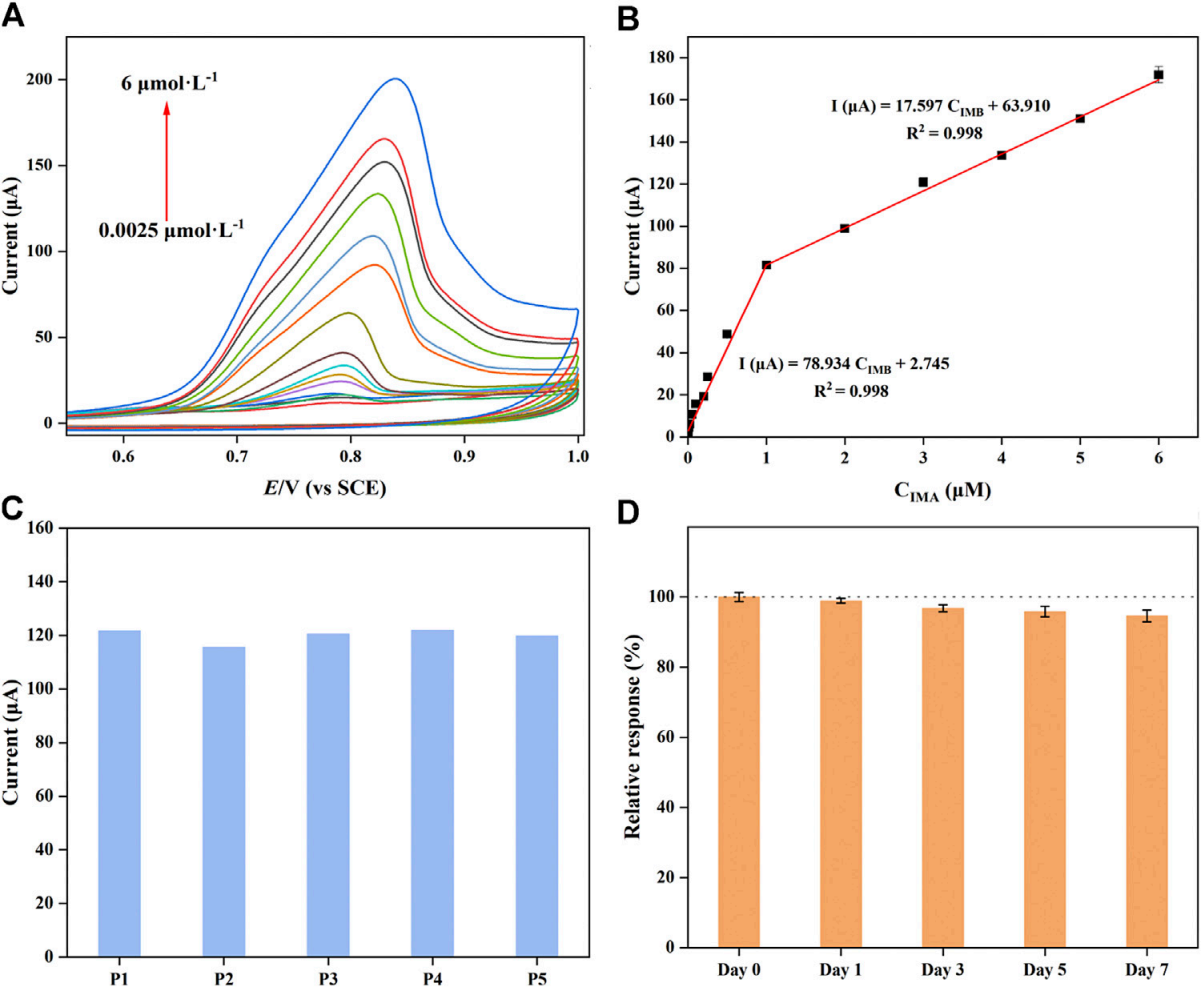
**(A)** Cyclic voltammograms and **(B)** calibration curves of CuMOF-AB/GCE at various concentrations (0.0025–6 μM) during IMB determination in PBS solution (pH = 7). **(C)** CV response of the five parallel electrodes to 3 μM IMB. **(D)** Stability of the fabricated sensors during a 7-day storage period.

Under the optimal experimental conditions, the analytical characteristics of the proposed sensor were studied by CV in PBS (0.1 M, pH = 7) during IMB determination. As shown in Figure 5A, as the IMB concentrations gradually increased, the intensity of the response peak current increased simultaneously. Although the catalytic current was positively proportional to concentration variations, the slopes of the calibration curve were different in low- and high-concentration regions. The two corresponding linear regression equations are shown in Figure 5B: I (μA) = 78.934 C_IMB_ + 2.745 (*R* ^2^ = 0.998, 2.5 nM−1.0 μM) and I (μA) = 17.597 C_IMB_ + 63.910 (*R* ^2^ = 0.998, 1.0–6.0 μM), respectively. These results were attributed to the IMB on the surface of the modified electrode forming monolayer coverage at low concentrations; in contrast, multilayers covered the surface at high concentrations. The limit of detection (LOD) was calculated with the following equation:

$$LOD=\frac{{3\sigma }_{blank}}{m}$$ (3)

where the signal-to-noise ratio is S/N = 3, σ_blank_ is the standard deviation of the blank and m is the slope value of the calibration curve; hence, the LOD was calculated as 1.7 nM. Compared with other already reported sensors for IMB determination (Table 1), the prepared electrochemical sensor exhibited a wider linear range and a generally lower LOD. And the electrode used for the sensor fabrication was GCE that had various advantages such as cost-effectiveness, admirable modification, facile accessibility and low background current (Jahani et al., 2022) when comparing with other electrodes. However, several reported IMB sensors not only gave higher DLs; but required tedious sample preparation and complicated process. In addition, the satisfactory sensitivity of the modified GCE was attributed to the high specific surface area of CuMOF and the excellent conductivity of AB. Accordingly, as-fabricated sensor could be potentially able to determine IMB in human serums.

**TABLE 1:** Comparison of various electrochemical sensor performances for IMB determination.

Sensors	Method	Linear range (μM)	LOD (nM)	Ref.
HMDE	SWV	0.019–1.9	5.6	Chen et al. (2014)
BDDE	DPV and CV	0.03–0.25	6.3	Brycht et al. (2016)
MWCNTs/SPCE	SWV and DPV	0.05–0.912	7.0	Rodríguez et al. (2018)
NiO-ZnO/MWCNT/GCE	DPV	0.015–2.0	2.4	Qian et al. (2021)
CuMOF-AB/GCE	CV and EIS	0.0025–6.0	1.7	This work

HMDE: hanging mercury drop electrode; BDDE: boron-doped diamond electrode; MWCNTs: multiwall carbon nanotubes; SPCE: screen-printed electrode; DPV: differential pulse voltammetry; SWV: square-wave voltammetry.

To demonstrate the high selectivity of the proposed sensors, a series of interference tests were performed to evaluate the response of IMB. In this experiment, the tolerance limit resulted in a relative oxidation current error of less than 5% for the determination of 3 μM IMB. Several organic and inorganic interferents, such as anions, cations, vitamins and amino acids, were used to evaluate the capability of anti-interference. According to the results shown in Supplementary Table S2, there was a negligible effect of the mentioned interfering species, indicating these interferences did not influence the responses of IMB determination. Therefore, the electrochemical sensor constructed by CuMOF-AB displayed a good capability of resisting interference and excellent selectivity for IMB determination.

To verify the precision and practicability of the electrochemical electrodes, the repeatability and long-term stability of CuMOF-AB/GCE were investigated. As shown in Figure 5C, five parallel modified electrodes (P1-P5) were constructed in the same batch and used to measure known concentrations of IMB by CV in 1 day. The relative standard deviation (RSD) of the oxidation current was calculated as 2.14%, illustrating that the designed strategy had good reproducibility. Additionally, the stability of the suggested sensors was demonstrated in-sequence by measuring the IMB signals of the current over a period of a week. The results are shown in Figure 5D. The maximal change in current was 5.35%, which proved that the structure of the AB and CuMOF framework had acceptable stability and a long service life.

### 3.7 Analytical application in real samples

Real sample analysis is essential to evaluate the practicality of the developed electrode in determining the analyte with an acceptable recovery. The applicability and reliability of the fabricated electrodes were investigated by using them for the determination of IMB in human blood serum samples. The samples were prepared by the process described in the **Experimental Section**. According to Table 2, the standard recovery rate of IMB in clinical serums ranged from 93.49% to 106.01% with an RSD between 1.04% and 9.43%. Meanwhile, the accuracy of IMB detection in patient serum samples was compared with LC-MS. The obtained results were summarized in Supplementary Table S3 based on an average of 3 measurements (n = 3), suggesting the acceptable precision of the proposed method. Thus, the fabricated novel sensor can be used as an effective method for measuring IMB anticancer drugs in real biological samples.

**TABLE 2:** Investigation of sensor applicability for IMB detection by standard addition method.

Sample	Added (μM)	CV method found (μM)	Recovery (%)	RSD (%) (n = 3)
1	0.5	0.5103	102.04	9.431
	1.0	0.9626	96.26	6.789
	2.0	1.9441	97.20	1.044
2	0.5	0.4654	93.49	4.312
	1.0	1.0601	106.01	2.107
	2.0	2.0169	100.85	2.305

## 4 Conclusion

In the present work, a new electrochemical sensor based on a CuMOF-AB nanocomposite was fabricated for the rapid and sensitive determination of IMB. CuMOF increased the attachment amount of IMB due to its excellent adsorbability, and AB enhanced electron transfer on the electrode surface due to its good conductivity. The effective CuMOF-AB nanocomposite exhibited improved electrocatalysis for IMB oxidation due to its greater conductivity and active catalytic sites. Under optimal conditions, the prepared sensor exhibited satisfactory selectivity, repeatability, good stability, a lower DL (1.7 nM) and a wider linear range (2.5 nM-1.0 μM and 1.0–6.0 μM, respectively). The CuMOF-AB-based sensor had a wider linear range and lower LOD than those reported for sensors in previous studies. In summary, a novel approach with excellent enforceability was developed for the sensitive and selective monitoring of IMB in biological samples.

## Data Availability

The original contributions presented in the study are included in the article/Supplementary Material, further inquiries can be directed to the corresponding authors.

## References

[B1] Abbasi A. R. Rizvandi M. Azadbakht A. Rostamnia S. (2016). Controlled uptake and release of imatinib from ultrasound nanoparticles Cu3(BTC)2 metal–organic framework in comparison with bulk structure. J. Colloid Interface Sci. 471, 112–117. 10.1016/j.jcis.2016.03.018 26994351

[B2] Abbasi A. R. Karimi M. Daasbjerg K. (2017). Efficient removal of crystal violet and methylene blue from wastewater by ultrasound nanoparticles Cu-MOF in comparison with mechanosynthesis method. Ultrason. Sonochem 37, 182–191. 10.1016/j.ultsonch.2017.01.007 28427622

[B3] Adiwidjaja J. Boddy A. V. McLachlan A. J. (2020). Physiologically-based pharmacokinetic predictions of the effect of curcumin on metabolism of imatinib and bosutinib: *In vitro* and *in vivo* disconnect. Pharm. Res. 37 (7), 128. 10.1007/s11095-020-02834-8 32529309

[B4] Azhar M. R. Abid H. R. Sun H. Periasamy V. Tadé M. O. Wang S. (2016). Excellent performance of copper based metal organic framework in adsorptive removal of toxic sulfonamide antibiotics from wastewater. J. Colloid Interface Sci. 478, 344–352. 10.1016/j.jcis.2016.06.032 27318714

[B5] Brycht M. Kaczmarska K. Uslu B. Ozkan S. A. Skrzypek S. (2016). Sensitive determination of anticancer drug imatinib in spiked human urine samples by differential pulse voltammetry on anodically pretreated boron-doped diamond electrode. Diam. Relat. Mater 68, 13–22. 10.1016/j.diamond.2016.05.007

[B6] Chen H. Wang X. Chopra S. Adams E. Van Schepdael A. (2014). Development and validation of an indirect pulsed electrochemical detection method for monitoring the inhibition of Abl1 tyrosine kinase. J. Pharm. Biomed. Anal. 90, 52–57. 10.1016/j.jpba.2013.11.022 24333704

[B7] Chen S. Wang C. Zhang M. Zhang W. Qi J. Sun X. (2020). N-doped Cu-MOFs for efficient electrochemical determination of dopamine and sulfanilamide. J. Hazard Mater 390, 122157. 10.1016/j.jhazmat.2020.122157 31999959

[B8] D’Avolio A. Simiele M. De Francia S. Ariaudo A. Baietto L. Cusato J. (2012). HPLC–MS method for the simultaneous quantification of the antileukemia drugs imatinib, dasatinib and nilotinib in human peripheral blood mononuclear cell (PBMC). J. Pharm. Biomed. Anal. 59, 109–116. 10.1016/j.jpba.2011.10.003 22036594

[B9] Diculescu V. C. Chiorcea-Paquim A.-M. Tugulea L. Vivan M. Oliveira-Brett A.-M. (2009). Interaction of imatinib with liposomes: Voltammetric and AFM characterization. Bioelectrochemistry 74 (2), 278–288. 10.1016/j.bioelechem.2008.10.003 19119081

[B10] Farag S. Verheijen R. B. Martijn Kerst J. Cats A. Huitema A. D. R. Steeghs N. (2017). Imatinib pharmacokinetics in a large observational cohort of gastrointestinal stromal tumour patients. Clin. Pharmacokinet. 56 (3), 287–292. 10.1007/s40262-016-0439-7 27435281

[B11] Feng J. Deng P. Xiao J. Li J. Tian Y. Wu Y. (2021). New voltammetric method for determination of tyrosine in foodstuffs using an oxygen-functionalized multi-walled carbon nanotubes modified acetylene black paste electrode. J. Food Compos Anal. 96, 103708. 10.1016/j.jfca.2020.103708

[B12] Foroughi M. M. Jahani S. Aramesh-Boroujeni Z. Vakili Fathabadi M. Hashemipour Rafsanjani H. Rostaminasab Dolatabad M. (2021). Template-free synthesis of ZnO/Fe3O4/Carbon magnetic nanocomposite: Nanotubes with hexagonal cross sections and their electrocatalytic property for simultaneous determination of oxymorphone and heroin. Microchem J. 170, 106679. 10.1016/j.microc.2021.106679

[B13] Gajski G. Gerić M. Domijan A. M. Golubović I. Garaj-Vrhovac V. (2019). Evaluation of oxidative stress responses in human circulating blood cells after imatinib mesylate treatment - implications to its mechanism of action. Saudi Pharm. J. 27 (8), 1216–1221. 10.1016/j.jsps.2019.10.005 31885482 PMC6921178

[B14] Gao Y. Zhou D. Xu Q. Li J. Luo W. Yang J. (2023). Metal–organic framework-mediated bioorthogonal reaction to immobilize bacteria for ultrasensitive fluorescence counting immunoassays. ACS Appl. Mater Interfaces 15 (4), 5010–5018. 10.1021/acsami.2c21350 36681942

[B15] Grossi M. Riccò B. (2017). Electrical impedance spectroscopy (EIS) for biological analysis and food characterization: A review. J. Sens. Sens. Syst. 6 (2), 303–325. 10.5194/jsss-6-303-2017

[B16] Guilhot F. Hughes T. P. Cortes J. Druker B. J. Baccarani M. Gathmann I. (2012). Plasma exposure of imatinib and its correlation with clinical response in the tyrosine kinase inhibitor optimization and selectivity trial. Haematologica 97 (5), 731–738. 10.3324/haematol.2011.045666 22315495 PMC3342976

[B17] Hammerich O. (2003). Electrochemical reactions and mechanisms in organic Chemistry, by james grimshaw. Electrochim Acta 48 (11), 1623–1624. 10.1016/S0013-4686(03)00086-0

[B18] Hassan Pour B. Haghnazari N. Keshavarzi F. Ahmadi E. Rahimian Zarif B. (2021). High sensitive electrochemical sensor for imatinib based on metal-organic frameworks and multiwall carbon nanotubes nanocomposite. Microchem J. 165, 106147. 10.1016/j.microc.2021.106147

[B19] Jahangiri–Dehaghani F. Zare H. R. Shekari Z. Benvidi A. (2022). Development of an electrochemical aptasensor based on Au nanoparticles decorated on metal–organic framework nanosheets and p-biphenol electroactive label for the measurement of aflatoxin B1 in a rice flour sample. Anal. Bioanal. Chem. 414 (5), 1973–1985. 10.1007/s00216-021-03833-3 35028689

[B20] Jahani S. Sedighi A. Toolabi A. Foroughi M. M. (2022). Development and characterization of La2O3 nanoparticles@snowflake-like Cu2S nanostructure composite modified electrode and application for simultaneous detection of catechol, hydroquinone and resorcinol as an electrochemical sensor. Electrochim Acta 416, 140261. 10.1016/j.electacta.2022.140261

[B21] Kralj E. Trontelj J. Pajič T. Kristl A. (2012). Simultaneous measurement of imatinib, nilotinib and dasatinib in dried blood spot by ultra high performance liquid chromatography tandem mass spectrometry. J. Chromatogr. B 903, 150–156. 10.1016/j.jchromb.2012.07.011 22857863

[B22] Kumari V. Pal Singh P. Kaushal S. (2022). Synthesis and applications of metal-organic frameworks and graphene-based composites: A review. Polyhedron 214, 115645. 10.1016/j.poly.2021.115645

[B23] Laviron E. (1979). General expression of the linear potential sweep voltammogram in the case of diffusionless electrochemical systems. J. Electroanal. Chem. Interfacial Electrochem 101 (1), 19–28. 10.1016/S0022-0728(79)80075-3

[B24] Li J. Xia J. Zhang F. Wang Z. Liu Q. (2018). An electrochemical sensor based on copper-based metal-organic frameworks-graphene composites for determination of dihydroxybenzene isomers in water. Talanta 181, 80–86. 10.1016/j.talanta.2018.01.002 29426545

[B25] Li G. Z. Fairweather M. Raut C. P. Wang J. (2022). Use of neoadjuvant imatinib to facilitate minimally invasive resection of gastric gastrointestinal stromal tumors. Ann. Surg. Oncol. 29 (11), 7104–7113. 10.1245/s10434-022-11891-9 35624191

[B26] Mioduszewska K. Dołżonek J. Wyrzykowski D. Kubik Ł. Wiczling P. Sikorska C. (2017). Overview of experimental and computational methods for the determination of the pKa values of 5-fluorouracil, cyclophosphamide, ifosfamide, imatinib and methotrexate. Trac. Trends Anal. Chem. 97, 283–296. 10.1016/j.trac.2017.09.009

[B27] Miura M. (2015). Therapeutic drug monitoring of imatinib, nilotinib, and dasatinib for patients with chronic myeloid leukemia. Biol. Pharm. Bull. 38 (5), 645–654. 10.1248/bpb.b15-00103 25947908

[B28] Mueller-Schoell A. Groenland S. L. Scherf-Clavel O. van Dyk M. Huisinga W. Michelet R. (2021). Therapeutic drug monitoring of oral targeted antineoplastic drugs. Eur. J. Clin. Pharmacol. 77 (4), 441–464. 10.1007/s00228-020-03014-8 33165648 PMC7935845

[B29] Özkan S. A. Uslu B. Zuman P. (2004). Electrochemical oxidation of sildenafil citrate (Viagra) on carbon electrodes. Anal. Chim. Acta 501 (2), 227–233. 10.1016/j.aca.2003.09.033

[B30] Peng B. Lloyd P. Schran H. (2005). Clinical pharmacokinetics of imatinib. Clin. Pharmacokinet. 44 (9), 879–894. 10.2165/00003088-200544090-00001 16122278

[B31] Qi C. Cai Q. Zhao P. Jia X. Lu N. He L. (2016). The metal-organic framework MIL-101(Cr) as efficient adsorbent in a vortex-assisted dispersive solid-phase extraction of imatinib mesylate in rat plasma coupled with ultra-performance liquid chromatography/mass spectrometry: Application to a pharmacokinetic study. J. Chromatogr. A 1449, 30–38. 10.1016/j.chroma.2016.04.055 27139217

[B32] Qian L. Durairaj S. Prins S. Chen A. (2021). Nanomaterial-based electrochemical sensors and biosensors for the detection of pharmaceutical compounds. Biosens. Bioelectron. 175, 112836. 10.1016/j.bios.2020.112836 33272868

[B33] Qu P. Han J. Qiu Y. Yu H. Hao J. Jin R. (2019). Huaier extract enhances the treatment efficacy of imatinib in Ik6+ Ph+ acute lymphoblastic leukemia. Biomed. Pharmacother. 117, 109071. 10.1016/j.biopha.2019.109071 31202171

[B34] Quan Y. Wang G. Jin Z. (2021). Tactfully assembled CuMOF/CdS S-Scheme heterojunction for high-performance photocatalytic H2 evolution under visible light. ACS Appl. Energy Mater 4 (8), 8550–8562. 10.1021/acsaem.1c01755

[B35] Rezvani Jalal N. Madrakian T. Afkhami A. Ghoorchian A. (2020). *In situ* growth of metal–organic framework HKUST-1 on graphene oxide nanoribbons with high electrochemical sensing performance in imatinib determination. ACS Appl. Mater Interfaces 12 (4), 4859–4869. 10.1021/acsami.9b18097 31908170

[B36] Rodríguez J. Castañeda G. Lizcano I. (2018). Electrochemical sensor for leukemia drug imatinib determination in urine by adsorptive striping square wave voltammetry using modified screen-printed electrodes. Electrochim Acta 269, 668–675. 10.1016/j.electacta.2018.03.051

[B37] Rodríguez Flores J. Berzas J. J. Castañeda G. Rodríguez N. (2003). Direct and fast capillary zone electrophoretic method for the determination of Gleevec and its main metabolite in human urine. J. Chromatogr. B 794 (2), 381–388. 10.1016/S1570-0232(03)00518-X 12954390

[B38] Roosendaal J. Groenland S. L. Rosing H. Lucas L. Venekamp N. Nuijen B. (2020). Determination of the absolute bioavailability of oral imatinib using a stable isotopically labeled intravenous imatinib-d8 microdose. Eur. J. Clin. Pharmacol. 76 (8), 1075–1082. 10.1007/s00228-020-02888-y 32430518 PMC7351863

[B39] Sánchez-López E. Marina M. L. Crego A. L. (2016). Improving the sensitivity in chiral capillary electrophoresis. Electrophoresis 37 (1), 19–34. 10.1002/elps.201500315 26434566

[B40] Schlichte K. Kratzke T. Kaskel S. (2004). Improved synthesis, thermal stability and catalytic properties of the metal-organic framework compound Cu3(BTC)2. Microporous Mesoporous Mater 73 (1), 81–88. 10.1016/j.micromeso.2003.12.027

[B41] Singh A. K. Jaiswal N. Tiwari I. Ahmad M. Silva S. R. P. (2023). Electrochemical biosensors based on *in situ* grown carbon nanotubes on gold microelectrode array fabricated on glass substrate for glucose determination. Microchim. Acta 190 (2), 55. 10.1007/s00604-022-05626-6 PMC9842592 36645527

[B42] Sun Y. Wang S. Cheng H. Dai Y. Yu J. Wu J. (2015). Synthesis of a ternary polyaniline@acetylene black-sulfur material by continuous two-step liquid phase for lithium sulfur batteries. Electrochim Acta 158, 143–151. 10.1016/j.electacta.2015.01.150

[B43] Taherizadeh M. Jahani S. Moradalizadeh M. Foroughi M. M. (2023). Synthesis of a dual-functional terbium doped copper oxide nanoflowers for high-efficiently electrochemical sensing of ofloxacin, pefloxacin and gatifloxacin. Talanta 255, 124216. 10.1016/j.talanta.2022.124216 36587425

[B44] Thommes M. Kaneko K. Neimark A. V. Olivier J. P. Rodriguez-Reinoso F. Rouquerol J. (2015). Physisorption of gases, with special reference to the evaluation of surface area and pore size distribution (IUPAC Technical Report). Pure Appl. Chem. 87 (9-10), 1051–1069. 10.1515/pac-2014-1117

[B45] Uslu B. Dogan B. Özkan S. A. Aboul-Enein H. Y. (2005). Electrochemical behavior of vardenafil on glassy carbon electrode: Determination in tablets and human serum. Anal. Chim. Acta 552 (1), 127–134. 10.1016/j.aca.2005.07.040

[B46] Wachholz Junior D. Deroco P. B. Kubota L. T. (2022). A copper-based metal-organic framework/reduced graphene oxide-modified electrode for electrochemical detection of paraquat. Microchim. Acta 189 (8), 278. 10.1007/s00604-022-05358-7 35829918

[B47] Walczak M. M. Dryer D. A. Jacobson D. D. Foss M. G. Flynn N. T. (1997). pH dependent redox couple: An illustration of the Nernst equation. J. Chem. Educ. 74 (10), 1195. 10.1021/ed074p1195

[B48] Wang F. Guo H. Chai Y. Li Y. Liu C. (2013). The controlled regulation of morphology and size of HKUST-1 by “coordination modulation method”. Microporous Mesoporous Mater 173, 181–188. 10.1016/j.micromeso.2013.02.023

[B49] Wang H. Jiang S. Pan J. Lin J. Wang J. Li M. (2022). Nanomaterials-based electrochemical sensors for the detection of natural antioxidants in food and biological samples: Research progress. Microchim. Acta 189 (9), 318. 10.1007/s00604-022-05403-5 35931898

[B50] Xu N. Ding Y. Ai H. Fei J. (2010). Acetylene black-ionic liquids composite electrode: A novel platform for electrochemical sensing. Microchim. Acta 170 (1), 165–170. 10.1007/s00604-010-0384-3

[B51] Yan Z. Zhang Z. Chen J. (2016). Biomass-based carbon dots: Synthesis and application in imatinib determination. Sens. Actuators B 225, 469–473. 10.1016/j.snb.2015.10.107

[B52] Yang X.-B. Zhu W. Qin K. Wang H.-Y. (2014). Preparation of lamellar carbon matrix for sulfur as cathode material of lithium-sulfur batteries. Electrochim Acta 143, 374–382. 10.1016/j.electacta.2014.07.080

[B53] Yola M. L. (2021). Sensitive sandwich-type voltammetric immunosensor for breast cancer biomarker HER2 detection based on gold nanoparticles decorated Cu-MOF and Cu2ZnSnS4 NPs/Pt/g-C3N4 composite. Microchim. Acta 188 (3), 78. 10.1007/s00604-021-04735-y 33569679

[B54] Zhang Y. He K. Han N. Wang L. Huang J. She H. (2023). Integration between CuMOF and g-C3N4 for effective suppressing charge recombination in photocatalytic peroxymonosulfate activation. J. Alloys Compd. 952, 170008. 10.1016/j.jallcom.2023.170008

[B55] Zheng H. Ying X. Wang W. Chen Z. Shao C. Zhou H. (2019). Study of sensitivity evaluation on ridgetail white prawn (Exopalaemon carinicauda) quality examination methods. Int. J. Food Prop. 22 (1), 942–951. 10.1080/10942912.2019.1617304

[B56] Zhou J. Li X. Yang L. Yan S. Wang M. Cheng D. (2015). The Cu-MOF-199/single-walled carbon nanotubes modified electrode for simultaneous determination of hydroquinone and catechol with extended linear ranges and lower detection limits. Anal. Chim. Acta 899, 57–65. 10.1016/j.aca.2015.09.054 26547493

