# Supplementary material for: Sensitivity enhancement of a Cu (II) metal organic framework-acetylene black-based electrochemical sensor for ultrasensitive detection of imatinib in clinical samples
Source: Front Chem. 2023 May 22;11:1191075. doi: 10.3389/fchem.2023.1191075 (PMC10239869; doi:10.3389/fchem.2023.1191075)
Supplement: Supplementary file 1 [file DataSheet1.docx]

**Supplementary Material**

**Sensitivity enhancement of a Cu(II) Metal Organic Framework-Acetylene Black-based electrochemical sensor for ultrasensitive detection of imatinib in clinical samples**

**Xuanming Xu^1^,** **Shun Li^1^, Xingwei Luan^1^, Chao Xuan^1^, Peng Zhao^1^, Tingting Zhou^1^*, Qingwu Tian^1^*, Deng Pan^1^***

^1^Department of Clinical Laboratory, The Affiliated Hospital of Qingdao University, No. 1677, Wutaishan Road, 266000 Qingdao, Shandong, China

***Correspondence:**

Deng Pan

dengpan@qdu.edu.cn

Qingwu Tian

tianqingwu@qdu.edu.cn

Tingting Zhou

zhoutingting@qdu.edu.cn

**2. Experimental section**

**2.1 Materials and Reagents**

N, N-dimethylformamide (DMF, 99.5%), methanol, ethanol and acetylene black (AB) were purchased from Aladdin Reagent (Shanghai Co. Ltd., China). Imatinib (IMB, ≥99%) was purchased from Shanghai Macklin Biochemical Co. Ltd. CuMOF (Cu_3_(BTC)_2_, HKUST-1) was obtained from Nanjing Xianfeng Nano Science and Technology Co. Ltd., China. Glucose, ascorbic acid, uric acid, dopamine were bought from Beijing Solarbio Science Technology Co. Ltd. MgSO_4_, FeSO_4_, CaCl_2_, FeCl_3_, ZnSO_4_, NaCl and KCl were purchased from Shanghai Hushi Laboratorial Equipment Co. Ltd. The 0.1 M different pH values of phosphate buffer solutions (PBSs) including NaH_2_PO_4_ and Na_2_HPO_4_ as supporting electrolyte, were configured using HCl or NaOH. The 0.1 mM IMB mother liquor was configured by dissolving pure IMB powder in metanol, and the experiment solutions were prepared daily from the stock solution. All of the analytical standard for interfering substances were analytical grade. Also the interfering agents concentrations of stock solutions were diluted to 0.03M. The extra all preparation of the aquesous solutions were used by deionized water.

**2.2 Apparatus and Measurements**

All electrochemical evaluations, including cyclic voltammetry (CV) and electrical impedance spectroscopy (EIS) were performed by the three-electrode workstation (CHI660C, Shanghai Chenhua Co., China). The reference electrode was a saturated calomel electrode (SCE), platinum (Pt) pillar electrode worked as auxiliary electrode, and the modified glassy carbon electrodes (GCEs, Φ=3mm) were used as working electrode. The determinations of pH value were performed with a precise pH-meter (PHS-3E, Shanghai INESA Scientific Instrument Co., Ltd, China).

**2.3 Preparing of the modified electrodes**

The GCEs were meticulously polished using 0.05 μm gamma alumina powder to obtain mirror-like surfaces. Then the electrodes were continuously rinsed by ethanol and deionized water respectively for 5 min in the ultrasonic bath. 1mg**·**ml^-1^ suspension was obtained after 10 mg of CuMOF precipitates were dispersed in 10 ml DMF ultrasonically for 30 min. AB (1mg**·**ml^-1^) suspension was also fabricated with the same conditions. 5 μL suspensions containing 1mg**·**ml^-1^ CuMOF and AB separately were dropped onto the cleaned electrode surfaces and then dried under the infrared lamp to prepare the CuMOF/GCE and AB/GCE.

To obtain the CuMOF-AB nanocomposite, different ratio of AB and CuMOF solids were ground in 0.5 ml DMF with a mortar and pestle for 5 min, then the mixture was centrifuged at 3000 rpm. After filtering, the resulting CuMOF-AB sediments were re-dispersed in DMF solvent using an ultrasonic probe. Next, the GCE surface was coated by homogenneous suspensions and exposed under infrared lamp to bake. The accomplished electrode was denoted as CuMOF-AB/GCE. Finally, the all preapred modification GCEs were characterized and used for the electrochemical experiment.

**2.4 The** **optimization of electrochemical** **procedure parameters**

In this experiment, electrochemical techniques were performed by CV and EIS methods. CV measurements were carried out with 500 rpm stirring rate and 7 min preconcentration time in 0.1 M PBS (pH=7) containing analytical substances. CV curves were recorded in the potential window of + 0.5V to + 1.1V at a scan rate of 100 mVs^−1^. Impedance spectra were obtained in 0.1 M KCl solution containing 5.0 mM [Fe(CN)_6_]^3−/4−^ in a range of frequencies from 100 kHz to 0.01 Hz with a 50 mV ac amplitude. All of the measured potentials in this literature were compared with those of calomel reference electrode and electrochemical procedures were carried out at room temperature.

**2.5 Preparing of** **the real samples**

The drug-free serum samples of healthy human were obtained from department of clinical laboratory and stored in a freezer (+4 ℃) before experiments. In order to remove the proteins in the samples, 1 mL methanol (as protein denaturant) and 0.5 mL serum were added into an Eppendorf tube. After adequate reaction, the serum samples were centrifuged at 14000 rpm for 5 min, then the acquired supernatant layer was taken and filtrated with a 0.22 μm microwell filtrator to receive purified serum samples. Next, 20 μL purified serum was added into 10 mL PBS (0.1M, pH=7) with certain levels of IMB (0.5 μM, 1μM, 2μM). Moreover, 0.5mL serums of different patients were diluted 10 times after methanol treatment. Finally, CuMOF-AB/GCEs were applied to each of the testing solutions, and the concentrations of IMB in the real samples were detected.

**3.1 Characterization of the materials**


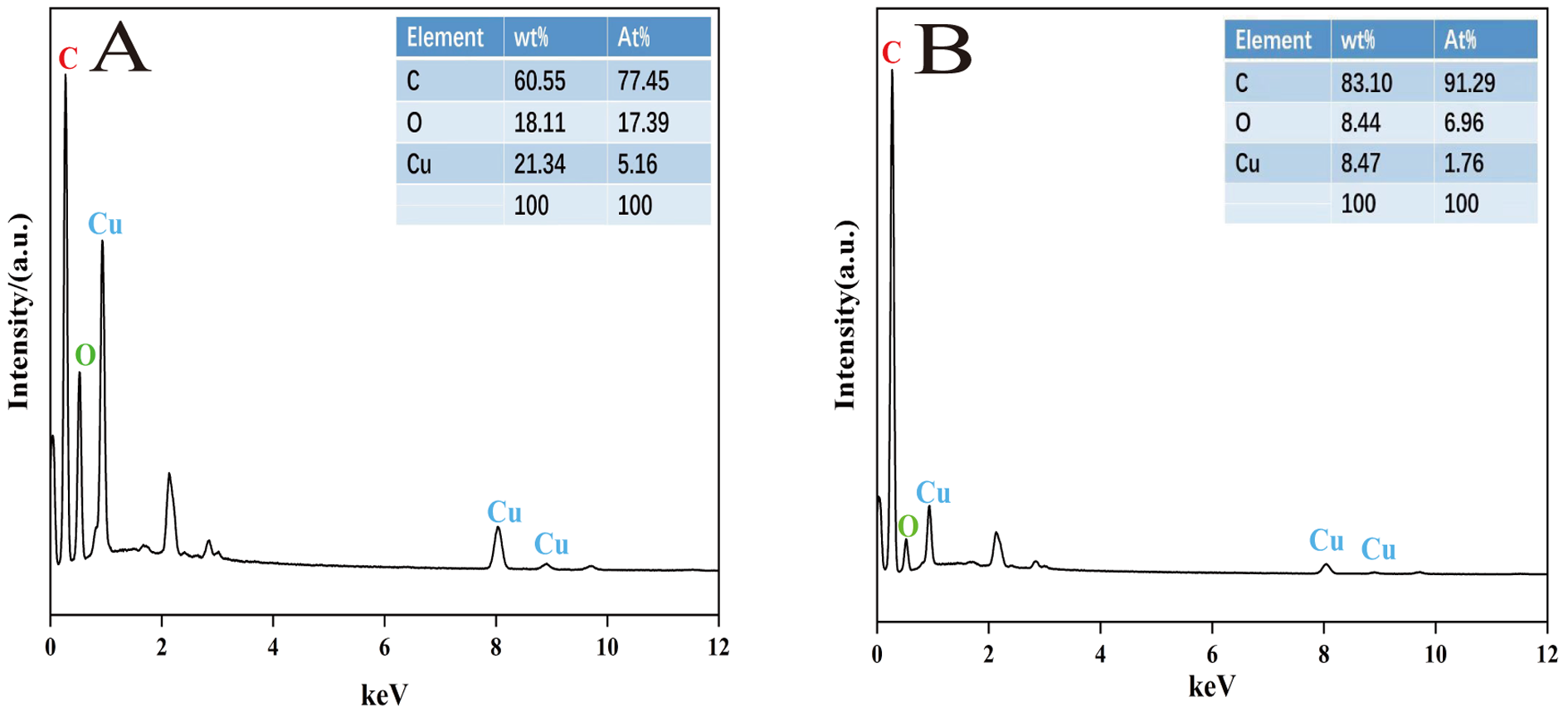


**Figure S1** The EDS characterization of the CuMOF (**A**) and CuMOF-AB (**B**).

**3.2 Electrochemical characterization of the modified electrodes**

**
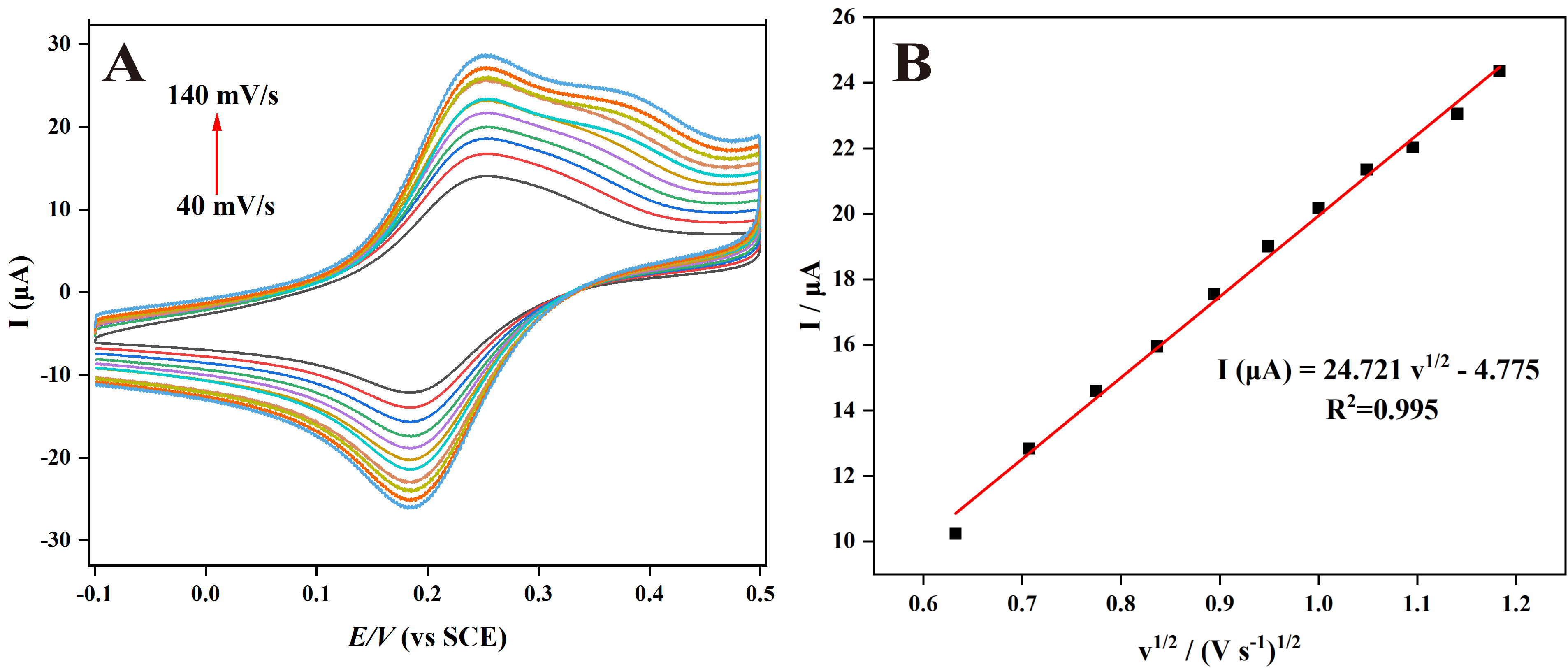
**

**Figure S2** CVs (**A**) of CuMOF-AB/GCE in the presence of 0.25 mM [Fe(CN)_6_]^3-^ solution in aqueous 0.1 M KCl at various scan rates (40-140 mV/s). The plot (**B**) of peak currents vs. υ^1/2^.

**3.4 Optimization of the** **analytical parameters**


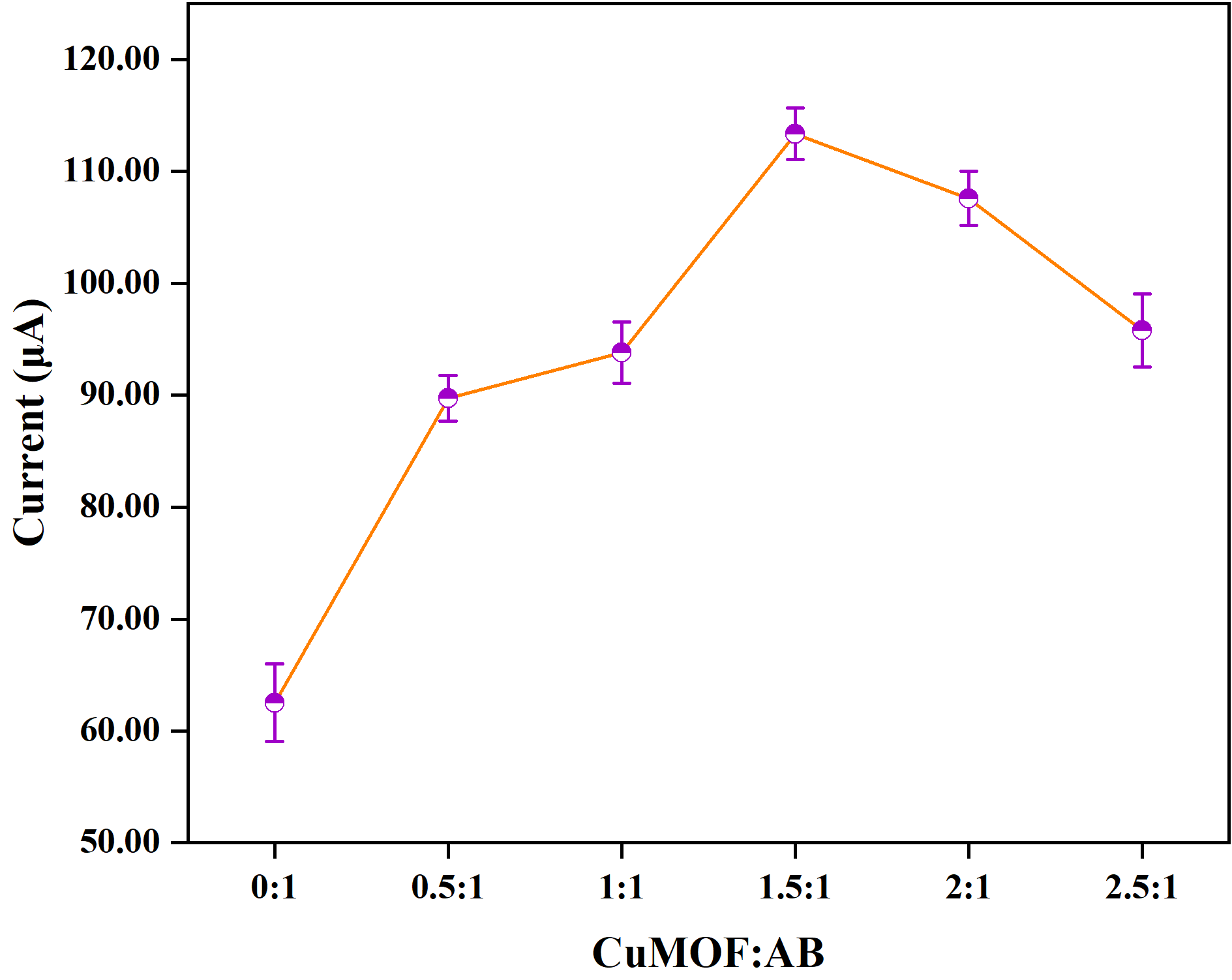


**Figure S3** The corresponding curves of the oxidation peak currents versus different mass ratio of CuMOF to AB.


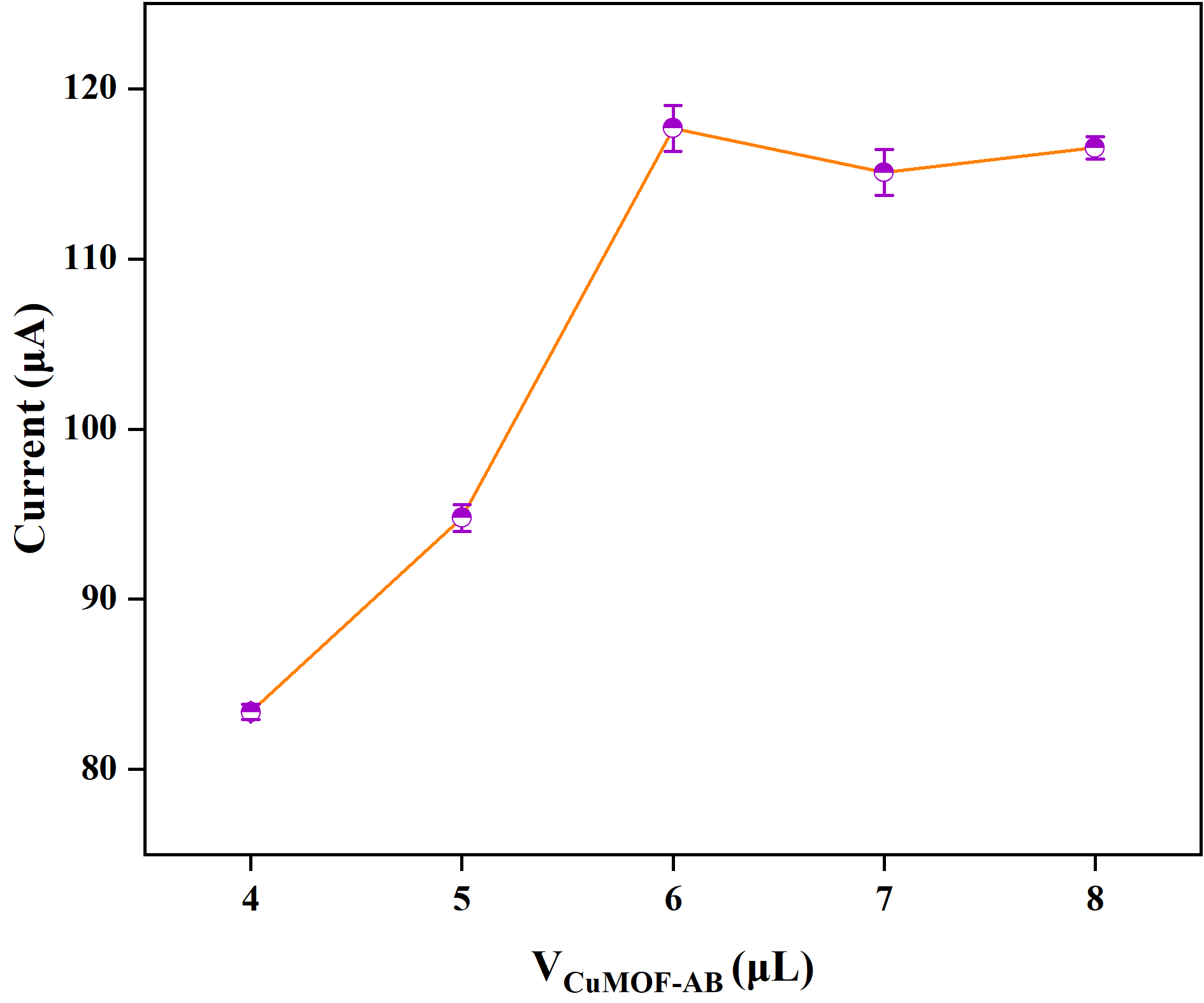


**Figure S4** The oxidation peak currents versus dropping volume of CuMOF-AB.


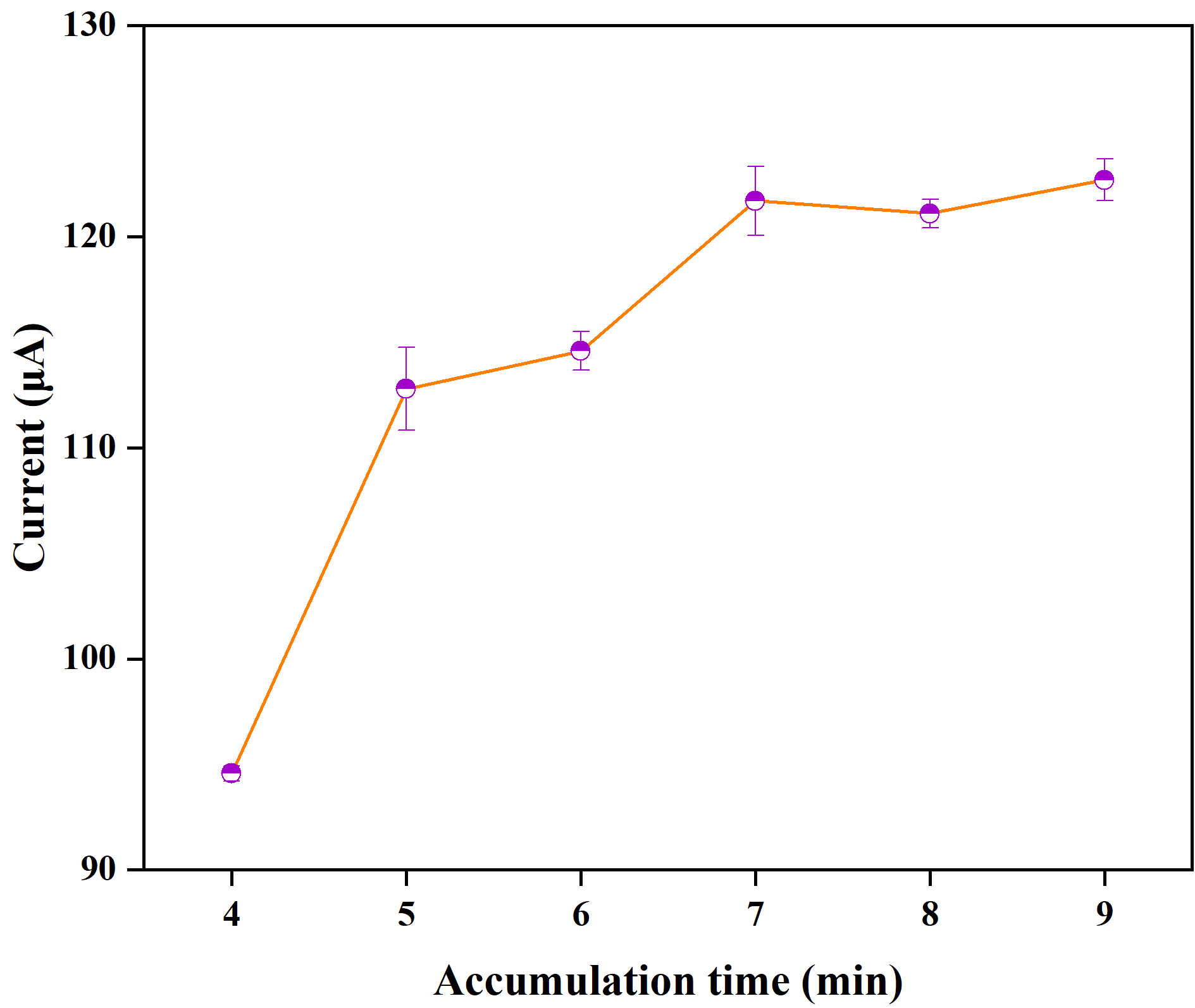


**Figure S****5** The oxidation peak currents versus IMB accumulation time in 0.1 M PBS (pH 7.0) containing 3 μM IMB.


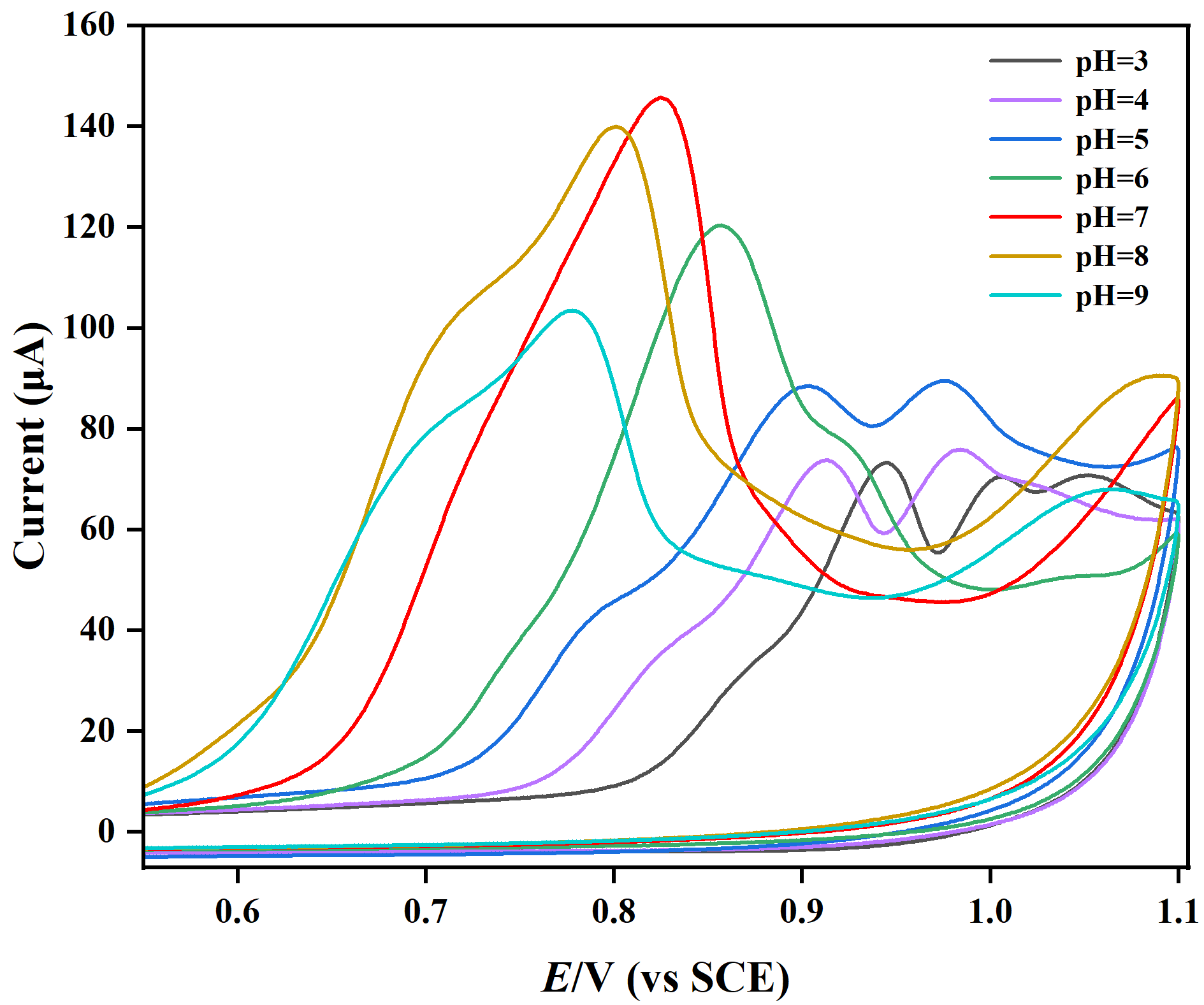


**Figure S6** CVs of CuMOF-AB modified electrode in 0.1 M PBS containing 3 μM IMB by varying pH values from 3.0 to 9.0 at 100 mV/s.


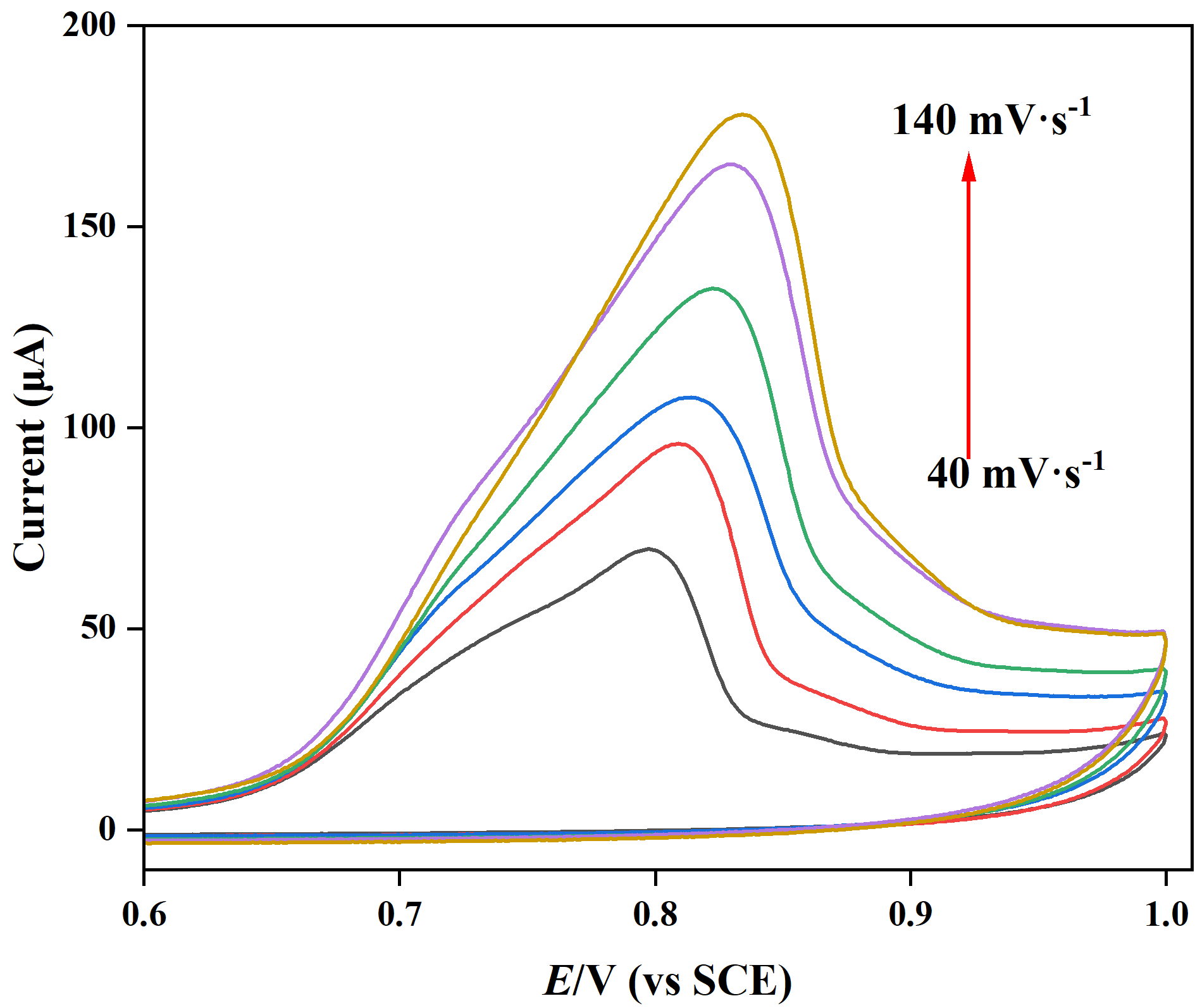


**Figure S7** CV curves recorded at CuMOF-AB/GCE in the presence of 3 μΜ IMB in PBS solution with variation scanning rate (40−140 mV/s).

**Table S1.** Specific surface area, pore volume and diameter of CuMOF and CuMOF-AB

| Material | Specific surface area (m^2^/g) | Pore volume (cc/g) | Pore diameter  (nm) |
| --- | --- | --- | --- |
| CuMOF | 448.993 | 0.6761 | 30.257  3.410 |
| CuMOF-AB | 228.443 | 0.4988 |  |

**Table S2.** Interference test of the fabricated sensors with different substances in 3μM IMB.

| Material | Interference level |
| --- | --- |
| K^+^, Na^+^, Cl^-^, Ca^2+^, Zn^2+^, Mg^2+^, Fe^3+^  Dopamine  Glucose, Uric acid  Ascorbic acid  Glycine, L-cysteine, Citric acid  Fe^2+^, SO_4_^2-^ | 1000  150  100  70  50  20 |

**Table S3. Comparison of different methods of IMB detection in human serum samples.**

| **Sample** | LC-MS method **Found (μM)** | CV method **Found (μM)** | **Recovery (%)** | **RSD (%) (n=3)** |
| --- | --- | --- | --- | --- |
| 1 | 1.2196 | 1.2154 | 99.66 | 1.777 |
| 2 | 1.4485 | 1.5012 | 103.64 | 6.451 |
| 3 | 1.8031 | 1.8604 | 103.18 | 3.586 |
| 4 | 2.2893 | 2.3409 | 102.25 | 4.351 |
| 5 | 2.9781 | 2.9851 | 100.24 | 3.438 |
| 6 | 4.0727 | 4.1196 | 101.15 | 5.889 |
